# Dual pathways linking mindfulness to life satisfaction and depression: the mediating roles of self-compassion and rumination in Chinese university students

**DOI:** 10.1186/s40359-025-02895-7

**Published:** 2025-05-27

**Authors:** Yunpeng Wu, Liping Qin, Xizheng Xu, Yu Tian, Zhe Jia

**Affiliations:** 1 School of Teacher Education, Dezhou University, Dezhou, China; 2 Department of Management, Hunan Police Academy, Changsha, China; 3 Department of Marxism, Qingdao University of Science and Technology, Qingdao, China; 4 Scientific Research Division, Qilu Medical University, Zibo, China

**Keywords:** Mindfulness, Self-compassion, Rumination, Life satisfaction, Depression, Sequential mediation, University students

## Abstract

**Objective:**

Mindfulness has been consistently linked to mental health benefits; however, the underlying mechanisms relating mindfulness to life satisfaction and depression remain underexplored. This study develops and empirically examines the Dual Pathways Mindfulness Model (DPMM), which posits that mindfulness is associated with mental health through sequential mechanisms involving self-compassion and rumination among university students.

**Methods:**

A cross-sectional survey involving 1,409 Chinese university students was conducted. Structural equation modeling (SEM) was used to test the hypothesized sequential mediation model. Indirect effects were examined using bias-corrected bootstrap confidence intervals.

**Results:**

Mindfulness was positively associated with life satisfaction (Effect = 0.080, *p* < 0.01) and negatively associated with depression (Effect = −0.180, *p* < 0.001). Self-compassion significantly mediated both associations, linking mindfulness to higher life satisfaction (Indirect effect = 0.057, 95% CI [0.05, 0.07]) and lower depression (Indirect effect = -0.033, 95% CI [−0.04, −0.03]). Rumination also served as a significant mediator for life satisfaction (Indirect effect = 0.067, 95% CI [0.02, 0.04]) and depression (Indirect effect = −0.064, 95% CI [ −0.07, −0.05]). Furthermore, a sequential mediation pathway was identified: higher mindfulness was associated with greater self-compassion, which was linked to lower rumination, ultimately associated with increased life satisfaction (Indirect effect = 0.020, 95% CI [0.01, 0.03]) and decreased depressive symptoms (Indirect effect = -0.039, 95% CI [−0.05, −0.03]).

**Conclusion:**

This study proposes and validates the DPMM, a novel model explaining how mindfulness relates to mental health through interconnected self-regulatory processes. By identifying self-compassion and rumination as sequential mediators, the findings offer theoretical insights into the psychological mechanisms linking mindfulness to enhanced well-being. While the cross-sectional design precludes causal claims, the results provide a foundational framework to guide future longitudinal studies and inform mental health promotion strategies grounded in mechanism-based understanding.

## Background

Mental health is increasingly recognized as a dual continuum encompassing both the absence of psychological distress (e.g., depression) and the presence of positive well-being (e.g., life satisfaction) [1, 2]. This dual-factor model is particularly critical for university students, a population navigating unique developmental challenges—academic pressure, identity formation, and future uncertainties—that elevate risks for depression while undermining life satisfaction [3, 4]. Research consistently links mindfulness to improved psychological outcomes. Yet, the mechanisms through which mindfulness is linked to both reduced psychological distress and enhanced well-being remain insufficiently understood, particularly within culturally specific contexts. Addressing this gap, the present study proposes and empirically tests the Dual Pathways Mindfulness Model (DPMM), which posits that mindfulness is associated with mental health through two interrelated processes: increased self-compassion and reduced rumination. By unraveling these pathways, we aim to clarify how mindfulness supports flourishing while mitigating psychological risk. Understanding how these mechanisms work is important for theory and for designing effective student mental health programs.

Mindfulness is defined as a non-judgmental awareness and acceptance of present-moment experiences, enabling individuals to respond with greater flexibility and reduced reactivity. It has been consistently associated with psychological well-being across diverse populations [5, 6]. A growing body of research demonstrates that higher mindfulness is associated with improved psychological adjustment—characterized by lower levels of depression and anxiety and greater life satisfaction—particularly among university students [7]. For example, mindfulness has been linked to increased happiness and reduced internalizing symptoms, with these relationships partially mediated by constructs such as a sense of purpose and behavioral activation [8]. Relatedly, mindfulness and self-esteem have been shown to positively correlate with life satisfaction in adolescent populations [9]. In the context of emerging adulthood, mindfulness and self-compassion have been negatively associated with depressive symptoms [10].

While the associations between mindfulness and mental health outcomes are well-documented, the underlying mechanisms remain underexplored in integrated frameworks. Although some studies have examined parallel or moderated mediation models [11], much of the earlier literature emphasized single mediators or direct bivariate associations, offering a relatively narrow view of how mindfulness relates to well-being. Recent evidence has highlighted the need for more complex frameworks that incorporate sequential and interactive mechanisms among psychological constructs [12]. For example, mindfulness may influence well-being through dynamic interactions between self-regulatory factors such as psychological flexibility, self-compassion, and rumination.

To clarify the mechanisms linking mindfulness to mental health, this study focuses on two central mediators: self-compassion and rumination. The present study is grounded in Self-Regulation Theory [13], which conceptualizes these constructs as distinct yet interrelated self-regulatory processes. According to this theory, individuals monitor and adjust their internal states to support goal-directed functioning. In this context, mindfulness is understood to facilitate adaptive self-regulation by enhancing self-compassion and reducing rumination. This framework offers a feedback-oriented perspective on how mindfulness may influence both emotional and cognitive functioning. Additionally, the Broaden-and-Build Theory [14] suggests that positive emotional states help expand individuals’ psychological resources over time. Together, these theories provide the conceptual basis for the Dual Pathways Mindfulness Model (DPMM), which posits that mindfulness supports mental health through two interrelated processes: cultivating self-compassion and reducing rumination. Specifically, mindfulness may contribute to the development of self-compassion by increasing emotional awareness and reducing self-critical tendencies, while simultaneously reducing ruminative thinking by promoting nonjudgmental attention and interrupting negative cognitive cycles. These pathways help clarify how mindfulness supports mental health through complementary self-regulatory processes.

Self-compassion, defined as offering kindness and understanding to oneself during difficult times [15], has consistently been associated with greater psychological well-being, including lower levels of depression and anxiety and higher life satisfaction [16–18]. By encouraging warmth and nonjudgment toward oneself during distress, self-compassion may foster positive experiences such as emotional safety and acceptance, which in turn facilitate psychological growth and resilience. Among university students, self-compassion has been shown to mediate the effects of stress and burnout on depressive symptoms, supporting its role in emotional regulation and mental health [19]. Meta-analytic and longitudinal evidence further suggests that self-compassion is a stable protective factor that may reduce vulnerability to psychopathology [20, 21].

Rumination, by contrast, involves persistent, repetitive negative thinking about distressing events or internal states [22] and is a well-established cognitive risk factor for depression [23–25]. It impairs problem-solving and prolongs negative affect [26]. Research has shown that mindfulness may help reduce rumination by promoting present-moment awareness and reducing automatic negative thinking [27, 28]. Reducing rumination, in turn, may lower cognitive vulnerability and interrupt maladaptive cycles of distress. Empirical evidence supports the integrative role of these two mediators in various populations. For example, rumination has been found to mediate the association between self-compassion and depression and anxiety [29]. Similarly, in parental populations, both self-compassion and types of rumination have been identified as pathways linking mindfulness to psychological outcomes [30]. These findings reinforce the relevance of integrating self-compassion and rumination within a unified framework to better understand how mindfulness relates to mental well-being.

Building on these theoretical foundations, we introduce the Dual Pathways Mindfulness Model (DPMM), which posits that mindfulness supports mental health through two interrelated self-regulatory processes: enhancing self-compassion and reducing rumination. These mechanisms represent complementary yet distinct pathways—one cultivating a kinder self-attitude, the other attenuating rigid negative thinking—both of which contribute to greater life satisfaction and fewer depressive symptoms. By integrating Self-Regulation Theory and the Broaden-and-Build Theory, the DPMM offers a comprehensive framework for understanding how mindfulness may concurrently promote psychological well-being and reduce emotional distress.

The current study aims to empirically test the proposed DPMM in a sample of Chinese university students. Examining these relationships within this cultural context helps assess the generalizability and cultural sensitivity of the model, contributing to a broader understanding of how mindfulness-related mechanisms function across diverse populations [31, 32]. Based on the DPMM framework, this study aims to examine the relationships among mindfulness, self-compassion, rumination, life satisfaction, and depression in a sample of Chinese university students. Investigating these associations in a non-Western context may offer culturally relevant insights into the mechanisms linking mindfulness and mental health outcomes [31, 32]. We hypothesize that mindfulness is positively associated with life satisfaction (H1a) and negatively associated with depression (H1b). We further propose that self-compassion mediates these associations: higher mindfulness is expected to be linked to greater self-compassion, which is in turn associated with higher life satisfaction (H2a) and lower depression (H2b). Additionally, we propose that rumination serves as another mediator: higher mindfulness is expected to be related to lower levels of rumination, which are associated with greater life satisfaction (H3a) and fewer depressive symptoms (H3b). Finally, we hypothesize a sequential mediation model (H4a and H4b) in which mindfulness is associated with greater self-compassion, which is in turn related to lower rumination, and this pathway is ultimately linked to increased life satisfaction and decreased depression.

## Materials and methods

### Participants

This study recruited a sample of university students from Shandong Province, China, using a cluster sampling method. Students were selected from multiple departments, with participants being randomly chosen from entire classes, as coordinated by faculty members (college counselors). A total of 1,520 students participated in the survey, with 1,409 valid questionnaires collected, resulting in a valid response rate of 92.70%.

Participants were primarily first-year students (approximately 90.77%, *n* = 1,279), supplemented by a smaller proportion of second-year students (approximately 9.23%, *n* = 130). First-year students were specifically targeted due to their transitional developmental stage, which is associated with increased vulnerability to psychological distress. A smaller group of second-year students was included to provide a preliminary perspective on these relationships once students have generally adapted to university life, thereby offering insights into the potential stability or change of psychological mechanisms examined. However, no formal comparison between first- and second-year students was conducted. The ages of the participants ranged from 18 to 22 years, with a mean age of 19.13 years (SD = 0.76). Most participants identified as Han Chinese (98.08%), whereas 27 participants (1.92%) were from various ethnic minority groups.

### Procedure

This study received approval from the institutional review board of Dezhou University and was conducted in accordance with the Declaration of Helsinki. Informed consent was obtained before the survey, ensuring confidentiality. The college counselor arranged the survey during the evening self-study period, and all students in the class participated. Participation was entirely voluntary, and students had the option to decline or withdraw from the survey at any time without penalty.

To ensure adequate statistical power, we conducted an a priori power analysis using G*Power 3.1. The analysis was based on the linear multiple regression: fixed model, R² deviation from zero setting, which is appropriate for estimating sample size in structural equation modeling (SEM). Assuming a medium effect size (f² = 0.15), α = 0.05, and power = 0.95, the analysis yielded a minimum required sample size of 686 participants. Our final valid sample of 1,409 students exceeded this requirement, ensuring sufficient statistical power for SEM and mediation analyses.

Participants completed an online survey via the Wenjuanxing platform in a password-protected session within a multimedia classroom. Participants completed the survey at their own pace, with a maximum time limit of 40 min for completion. Participants were rewarded with moral education activity points (a form of academic credit for extracurricular activities in Chinese universities) for their involvement in the study. The survey measured mindfulness, self-compassion, rumination, life satisfaction, and depression using standardized instruments. Sum scoring was applied for all scales, where higher scores indicated higher levels of the respective constructs. Items were presented in a fixed order (not randomized) to maintain scale integrity. There were no missing data, as participants were required to complete all items before submission. Those completed too quickly (shorter than 5 min) were excluded.

### Instruments

#### Mindful Attention Awareness Scale (MAAS)

The Chinese version of the Mindful Attention Awareness Scale (MAAS) was translated and revised by Chen et al. [33]. This unidimensional scale consists of 15 items that assess the degree to which individuals are aware of and attentive to the present moment in their daily lives. For example, items include statements such as “I tend to walk quickly to get where I’m going without paying attention to what I experience along the way.” Responses are recorded on a 6-point Likert scale ranging from “almost always” (1) to “almost never” (6), with higher scores indicating higher levels of mindfulness. The MAAS demonstrated good internal consistency in the current study, with a Cronbach’s α coefficient of 0.895.

#### Ruminative Responses Scale (RRS)

The Chinese version of the Ruminative Responses Scale (RRS), translated and revised by Han et al. [34], was used to measure rumination. The scale includes 22 items across three dimensions: symptom rumination, reflective pondering, and brooding. The participants responded to statements that described their focus on self, depressive symptoms, and related causes and consequences, such as “I often analyze recent events to understand why I feel depressed.” Responses are measured on a 4-point Likert scale ranging from “almost never” (1) to “always” (4), with higher scores indicating higher levels of rumination. In this study, the total Cronbach’s α coefficient for the RRS was 0.939, with the three subscales showing internal consistency coefficients of 0.917, 0.807, and 0.751, respectively, indicating good reliability.

#### Self-Compassion Scale– Short Form (SCS-SF)

This study employed the Chinese version of the 12-item short form of the Self-Compassion Scale (SCS-SF). The reliability and validity of this short form have been well established in Chinese adolescent populations [35]. While the original Self-Compassion Scale comprises six subcomponents, the short form used in this study adopts a three-factor structure—self-kindness, common humanity, and mindfulness—which has been empirically validated in Chinese samples. These three dimensions reflect key aspects of a compassionate self-attitude. Although one of the subscales is labeled “mindfulness,” it specifically refers to a nonjudgmental and accepting attitude toward one’s own suffering, which conceptually differs from the present-focused awareness assessed by the MAAS used as the independent variable. Therefore, conceptual and measurement overlap between the two constructs is limited. Participants rated each item on a 5-point Likert scale ranging from “almost never” (1) to “almost always” (5), with higher scores indicating greater self-compassion. In the present sample, the SCS-SF demonstrated good internal consistency (α = 0.813), with subscale coefficients of 0.701, 0.732, and 0.848, respectively.

#### Satisfaction with Life Scale (SWLS)

The Satisfaction with Life Scale (SWLS), developed by Diener et al. [36] and revised by Xiong and Xu [37] into Chinese, was used to measure participants’ life satisfaction. This scale consists of 5 items, each rated on a 7-point Likert scale ranging from “strongly disagree” (1) to “strongly agree” (7). Higher scores indicate greater satisfaction with life. In this study, the SWLS demonstrated good internal consistency, with a Cronbach’s α coefficient of 0.889.

#### Depression scale

The Center for Epidemiologic Studies Depression Scale (CES-D) was used to assess depressive symptoms in participants. This study employed the 10-item short version of the CES-D [38], which has been validated in Chinese samples [39]. Each item is rated on a 4-point Likert scale ranging from “rarely or none of the time” (0) to “most or all of the time” (3). Higher scores indicate more severe depressive symptoms. The CES-D demonstrated adequate internal consistency in this study, with a Cronbach’s α coefficient of 0.862.

### Data analysis

The data were analyzed using SPSS 27.0 for descriptive analyses and t-tests, and Mplus 7.0 for structural equation modeling (SEM). Descriptive statistics and correlation analyses were conducted in SPSS to examine relationships between variables, whereas independent samples t-tests were performed to assess gender and grade differences in the primary study variables.

For the mediation analysis, Mplus was employed to test a sequential mediation model exploring the indirect relationships between mindfulness and the two dependent variables—life satisfaction and depression—through self-compassion and rumination. The model treated mindfulness as the independent variable, self-compassion and rumination as mediators, and life satisfaction and depression as dependent variables, while controlling for gender and grade. The structural equation model specified a correlation between life satisfaction and depression, based on their well-documented inverse relationship in the literature as indicators of positive well-being and psychological distress, respectively.

The goodness-of-fit for each model was evaluated via several indices: χ² (chi-square test), df (degrees of freedom), CFI (comparative fit index), TLI (Tucker‒Lewis index), and RMSEA (root mean square error of approximation). Following the recommendations of prior research, model fit was considered satisfactory if χ²/df ≤ 5, CFI and TLI ≥ 0.95, and RMSEA ≤ 0.05 [40]. To estimate indirect effects and assess their significance, a bias-corrected bootstrap approach with 5,000 samples was used. Bootstrapping is widely recommended for mediation analysis as it provides robust confidence intervals without requiring normality assumptions [41].

## Results

### Common method bias test

To address potential common method bias [42], procedural and statistical remedies were implemented. Procedurally, participant anonymity was strictly ensured to reduce evaluation apprehension, and reverse-scored items were incorporated into the scales to minimize acquiescence bias. Statistically, Harman’s single-factor test revealed ten factors with eigenvalues greater than one, accounting for 58.15% of the variance, with the first factor explaining only 28.36% of the variance—below the critical 40% threshold. These findings indicate that common method bias is not a significant concern in this study.

### Descriptive statistics and correlation analysis of the variables

Independent samples t tests were used to assess gender and grade differences in life satisfaction, rumination, self-compassion, and depression (Table 1). Compared with female students, male students presented significantly higher rumination levels (*p* = 0.027), whereas no gender differences were found in life satisfaction, self-compassion, or depression (*p* > 0.05). Compared with second-year students, first-year students presented significantly greater self-compassion (*p* = 0.006) and lower depression (*p* = 0.049), with no grade differences in life satisfaction and rumination (*p* > 0.05).

Pearson correlation analysis (Table 2) indicated that mindfulness was positively correlated with self-compassion and life satisfaction and negatively correlated with rumination and depression. Self-compassion was positively associated with life satisfaction and negatively associated with rumination and depression. Rumination was negatively correlated with life satisfaction and positively correlated with depression. These results reflect significant associations among the key variables, supporting the plausibility of the hypothesized mediation model.

**Table 1:** Comparison of key variables by gender and grade level

Variable	Gender difference					Grade difference				
	Male (M ± SD)	Female (M ± SD)	t	*p*	Cohen’s d	Year 1 (M ± SD)	Year 2 (M ± SD)	t	*p*	Cohen’s d
Life satisfaction	24.64 ± 6.42	24.01 ± 6.02	1.86	0.063	0.10	24.27 ± 6.26	24.15 ± 5.39	0.20	0.845	0.02
Rumination	46.29 ± 12.07	44.86 ± 11.72	2.22	0.027	0.12	45.24 ± 11.82	47.14 ± 12.24	−1.74	0.082	−0.16
Self-compassion	42.26 ± 6.49	41.67 ± 6.94	1.59	0.113	0.09	42.06 ± 6.86	40.33 ± 5.68	2.78	0.006	0.26
Depression	13.66 ± 5.83	13.36 ± 5.48	0.99	0.319	0.05	13.38 ± 5.65	14.40 ± 5.21	−1.97	0.049	−0.19

Note. Cohen’s d values represent the effect size, with guidelines for interpretation as follows: Small effect (d ≈ 0.20), Medium effect (d ≈ 0.50), and Large effect (d ≈ 0.80).

**Table 2:** Descriptive statistics and correlations among key variables (*N* = 1409)

Variable	Mean	SD	1	2	3	4	5
1. Mindfulness	60.87	13.06	1	0.446^***^	−0.471^***^	0.306^***^	−0.493^***^
2. Self-compassion	41.90	6.77		1	−0.529^***^	0.431^***^	−0.518^***^
3. Rumination	45.41	11.87			1	−0.410^***^	0.679^***^
4. Life satisfaction	24.25	6.18				1	−0.472^***^
5. Depression	13.48	5.62					1

Note. ***p < 0.001. Correlation coefficients: Small effect (*r* < 0.30), Medium effect (0.30 ≤ *r* < 0.50), Large effect (*r* ≥ 0.50)

Given the significant correlations observed, gender and grade were included as control variables in the structural model to account for their potential influence on the main outcomes. Variance inflation factor (VIF) values ranged from 1.38 to 1.53, well below the commonly accepted threshold of 5, indicating no multicollinearity concerns and confirming that the data met the assumptions for further structural modeling.

### Testing of hypotheses

The structural equation model (SEM) developed to explore the relationships between mindfulness, self-compassion, rumination, depression, and life satisfaction demonstrated a strong overall fit, with excellent fit indices (χ² = 16.01, df = 4, CFI = 0.995, TLI = 0.978, RMSEA = 0.046). These fit indices indicate that the hypothesized model fits the observed data well, suggesting that the proposed relationships are adequately supported.

**Fig. 1:**
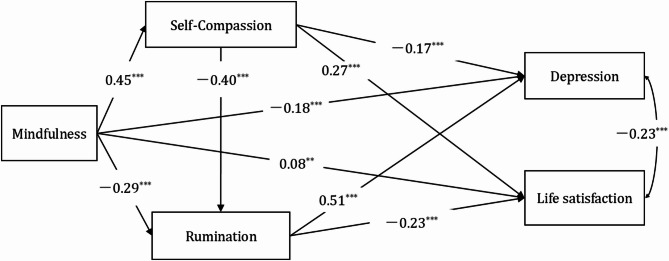
Path model of associations between mindfulness, self-compassion, rumination, depression, and life satisfaction Note: *N* = 1409. For visual clarity, control variables (gender and grade level) and their associated regression paths to all endogenous variables are not displayed. The model includes a specified covariance between life satisfaction and depression (standardized estimate = − 0.23), as shown. All path coefficients are standardized. ** *p* < 0.01, ***p < 0.001

As expected, life satisfaction and depression were significantly negatively correlated in the model (standardized estimate = − 0.23, *p* < 0.001), supporting their conceptualization as inverse indicators of psychological well-being. Including this covariance accounts for shared variance between the two outcome variables, ensuring that the indirect effects of mindfulness via self-compassion and rumination are estimated above and beyond their overlap. This covariance was specified in the model and is reflected in Fig. 1, although control variables (gender and grade level) are not shown for visual clarity.

The results showed several significant associations, supporting the hypothesized model (Fig. 1). Mindfulness was positively associated with self-compassion (β = 0.45, *p* < 0.001) and life satisfaction (β = 0.08, *p* < 0.01), and negatively associated with rumination (β = − 0.29, *p* < 0.001) and depression (β = − 0.18, *p* < 0.001). These findings indicate that individuals with higher mindfulness tend to report greater self-compassion and life satisfaction, and lower rumination and depressive symptoms. The standardized coefficients indicate moderate to strong effects, particularly in the case of self-compassion and rumination, supporting the dual impact proposed in Hypotheses H1a and H1b.

**Table 3:** Regression coefficients for mediating role paths

Effect Type	Path	Effect	SE	*p*	95% Confidence Intervals
Depression					
Indirect Effect	Mindfulness → Self-Compassion → Depression	−0.033	0.005	< 0.001	[−0.04, −0.02]
Indirect Effect	Mindfulness → Rumination → Depression	−0.064	0.006	< 0.001	[−0.07, −0.05]
Indirect Effect	Mindfulness → Self-Compassion → Rumination → Depression	−0.039	0.004	< 0.001	[−0.05, −0.03]
Life Satisfaction					
Indirect Effect	Mindfulness → Self-Compassion → Life Satisfaction	0.057	0.008	< 0.001	[0.05, 0.07]
Indirect Effect	Mindfulness → Rumination → Life Satisfaction	0.067	0.007	< 0.001	[0.02, 0.04]
Indirect Effect	Mindfulness → Self-Compassion → Rumination → Life Satisfaction	0.020	0.003	< 0.001	[0.01, 0.03]

Note. Indirect effects represent estimated effect sizes derived from bias-corrected bootstrap analyses. Confidence intervals indicate the 95% range within which the true effect size is expected to fall. Indirect effects reflect the mediating roles of self-compassion and rumination in the relationships between mindfulness and mental health outcomes (depression and life satisfaction)

Self-compassion was positively associated with life satisfaction (β = 0.27, *p* < 0.001) and negatively associated with depression (β = − 0.17, *p* < 0.001), while rumination was negatively associated with life satisfaction (β = − 0.23, *p* < 0.001) and positively associated with depression (β = 0.51, *p* < 0.001). These associations support the mediating roles of self-compassion and rumination in linking mindfulness with mental health indicators.

The mediation analysis (Table 3) supported that both self-compassion and rumination partially explained the associations between mindfulness and the outcome variables. Specifically, mindfulness was indirectly associated with lower depression via self-compassion (95% CI: [–0.04, − 0.02]) and rumination (95% CI: [–0.07, − 0.05]); and with higher life satisfaction via self-compassion (95% CI: [0.05, 0.07]) and rumination (95% CI: [0.02, 0.04]). These findings suggest two complementary pathways: greater mindfulness was related to higher self-compassion, which in turn correlated with higher life satisfaction and lower depressive symptoms; while greater mindfulness was also related to lower rumination, which in turn was associated with better mental health outcomes. These results support Hypotheses H2a, H2b, H3a, and H3b.

In addition, significant sequential mediation effects were observed (Table 3), supporting H4a and H4b. Specifically, mindfulness was associated with increased self-compassion, which in turn was associated with reduced rumination, ultimately relating to higher life satisfaction (95% CI: [0.01, 0.03]) and lower depression (95% CI: [–0.05, − 0.03]). These findings underscore the importance of examining interconnected mediators in understanding the psychological processes underlying mindfulness.

## Discussion

This study proposed and tested the Dual Pathways Mindfulness Model (DPMM), a novel framework grounded in Self-Regulation Theory and the Broaden-and-Build Theory. Using a sample of Chinese university students, we examined how dispositional mindfulness is linked to mental health through two interrelated processes: increased self-compassion and reduced rumination. The findings indicate that mindfulness is concurrently associated with greater life satisfaction and fewer depressive symptoms via these complementary mechanisms. By elucidating these regulatory processes, the study contributes to a more integrated understanding of how mindfulness relates to mental health. Furthermore, validating the DPMM in a Chinese cultural context provides initial support for its cross-cultural relevance.

### Mediating mechanisms of self-compassion and rumination

The findings of this study underscore self-compassion as a key mediating variable in the relationship between mindfulness and mental health outcomes. Specifically, greater dispositional mindfulness was associated with higher levels of self-compassion, which in turn related positively to life satisfaction and negatively to depressive symptoms. In line with the broaden-and-build theory, self-compassion fosters positive emotional states that expand individuals’ cognitive and emotional capacities, thereby building long-term psychological resources.

A growing body of literature supports the mediating role of self-compassion in emotional regulation and psychological well-being. Prior studies have shown that self-compassion helps buffer the effects of stress, depression, and trauma by promoting adaptive emotional responses and reducing self-critical tendencies [43]. Moreover, self-compassion enhances life satisfaction and reduces depressive symptoms through both affective and cognitive pathways, particularly by facilitating positive automatic thoughts that reshape emotional experiences [44]. It also acts as a resilience factor, enabling individuals to cope more effectively with adversity by reinforcing emotional stability and nurturing self-kindness [45]. Consistent with these findings, our study demonstrates that self-compassion plays a central role in linking mindfulness with improved psychological well-being. Meta-analyses have further confirmed that self-compassion is associated with reduced levels of rumination, anxiety, and depression, as well as greater emotional stability and positive affect [46].

In contrast, rumination emerged as another critical mediator, aligning with prior research that links it to sustained negative mood and elevated depressive symptomatology [47]. A wealth of empirical evidence indicates that rumination amplifies psychological distress by prolonging negative mood states, impairing problem-solving capacity, and increasing sensitivity to environmental stressors [48]. Individuals with a ruminative thinking style often show heightened emotional reactivity, which intensifies symptoms of both depression and anxiety [49, 50]. Cognitive studies have also demonstrated that repeated retrieval of negative memories diminishes mental flexibility and disrupts attentional control [51].

Mindfulness appears to mitigate these maladaptive cognitive tendencies by interrupting automatic negative thought patterns and promoting more adaptive cognitive styles [52]. This shift in mental processing reduces the likelihood of engaging in repetitive, self-focused negative thinking and supports overall psychological functioning. Rather than viewing self-compassion and rumination as strictly emotional or cognitive mechanisms, it is more appropriate to conceptualize them as multifaceted self-regulatory processes involving both affective and cognitive components—such as emotional awareness, self-reflection, and attentional control. In this study, self-compassion and rumination functioned as central pathways through which mindfulness was associated with mental health outcomes. These findings underscore the role of mindfulness in strengthening interconnected psychological capacities that regulate thoughts and emotions in a dynamic, integrated manner.

### Sequential mediating role of self-compassion and rumination

A central contribution of this study is the conceptualization and empirical validation of a sequential mediation model that clarifies how mindfulness may be linked to better mental health outcomes through the interplay of two key intrapersonal mechanisms—self-compassion and rumination. Specifically, mindfulness was associated with greater self-compassion, which in turn was linked to lower levels of rumination—ultimately contributing to increased life satisfaction and reduced depression. This pathway suggests that fostering a kinder, more accepting self-attitude may help interrupt repetitive negative thinking and promote psychological well-being.

The model is informed by self-regulation theory, which highlights the importance of monitoring and adjusting internal states to maintain goal-directed functioning. Within this framework, reducing habitual thought patterns such as rumination can support emotional recovery and enhance psychological functioning [53]. Self-compassion has been shown to play a protective role by fostering self-acceptance, reducing self-criticism, and promoting adaptive coping in the face of stress and negative affect [46]. Additionally, it facilitates the generation of positive automatic thoughts that buffer against depressive symptoms and enhance emotional resilience [44].These effects may indirectly reduce rumination by weakening harsh self-evaluations and increasing emotional clarity, thereby limiting the escalation of negative thought cycles [49, 50].

Rumination, characterized by persistent and repetitive focus on distress and its causes, is a known risk factor for the maintenance and intensification of depressive symptoms [47]. It impairs cognitive flexibility and problem-solving abilities [51], making it more difficult for individuals to disengage from negative thoughts. Mindfulness may counteract these effects by promoting non-judgmental awareness and decentering from negative internal experiences, thereby reducing ruminative tendencies [52].

By integrating these mechanisms into a coherent sequence, the DPMM advances theoretical understanding of how mindfulness may support psychological functioning. The model highlights how self-compassion may initiate upward regulatory change that subsequently dampens maladaptive cognitive patterns, reinforcing the relevance of examining their interaction rather than treating them in isolation. This framework provides a foundation for future work on dynamic self-regulation processes in mental health.

### Strengths of the current study

A central strength of this study lies in the development and empirical evaluation of the Dual Pathways Mindfulness Model (DPMM)—a novel framework that integrates Self-Regulation Theory and the Broaden-and-Build Theory to explain how mindfulness is associated with both enhanced life satisfaction and reduced depressive symptoms. By identifying self-compassion and rumination as two interrelated self-regulatory processes, the model offers an integrative application of the dual-factor model of mental health [1, 2], which emphasizes understanding both well-being and psychological distress within a unified framework.

Beyond identifying parallel mediators, this study further contributes to the literature by testing a sequential pathway in which greater mindfulness is linked to higher self-compassion, which in turn relates to lower rumination. This sequential association suggests a possible “positive regulatory cascade,” wherein cultivating a more supportive self-attitude may help reduce maladaptive thought patterns—such as repetitive negative thinking (e.g., positive automatic thoughts [44]; rumination [52])—and enhance overall psychological functioning. While prior research has examined multi-mediator models, few studies have investigated how these mechanisms operate in tandem and build upon each other.

A third notable strength lies in the study’s cultural context. By testing the DPMM among Chinese university students, the research addresses a key gap in the mindfulness literature, which has been largely dominated by Western samples. The findings suggest that self-compassion remains a robust psychological resource even in collectivist contexts that value modesty and self-criticism [45, 46], while rumination—though widely regarded as maladaptive—may be influenced by culturally shaped emotion regulation norms, such as restraint and relational harmony [54].

### Limitations and future directions

Despite offering valuable insights, this study is subject to several limitations that warrant consideration. First, the cross-sectional design prevents causal inference, limiting our ability to determine the directionality or temporal ordering of relationships among mindfulness, self-compassion, rumination, life satisfaction, and depression. Future research should employ longitudinal or experimental designs to examine the dynamic and potentially reciprocal nature of these psychological processes over time.

Second, the reliance on self-report measures may introduce biases such as social desirability and recall errors, potentially affecting the validity of the findings [42]. To enhance methodological robustness, future studies could incorporate multimethod approaches—such as behavioral observations, peer reports, or physiological indicators (e.g., heart rate variability)—to triangulate subjective data.

Third, although self-compassion and rumination were identified as significant mediators in this study, their roles may be influenced by cultural values and regulatory norms. For instance, in East Asian dialectical cultures, indirect self-regulation (e.g., reducing self-judgment) may be more culturally congruent than overt self-kindness [55], and cultural attribution styles (e.g., heightened self-monitoring or self-doubt) may intensify rumination’s psychological impact [54]. These findings suggest that while the DPMM’s mechanisms may hold cross-cultural relevance, their expression and function likely vary across sociocultural contexts, requiring further cultural adaptation and validation.

Finally, the sample was limited to Chinese university students, which may restrict the generalizability of findings to other age groups or cultural populations. Although the psychological mechanisms examined (e.g., self-compassion, rumination) are theorized to be universal, contextual factors—such as educational pressures, relational norms, or spiritual practices—may shape how mindfulness is experienced and enacted. Future research should validate the DPMM in diverse populations and explore culture-specific mediators (e.g., gratitude in collectivist settings or autonomy in individualistic cultures), as the psychological mechanisms of mindfulness may vary across different educational, social, and spiritual contexts [56].

## Conclusions

This study developed and tested the Dual Pathways Mindfulness Model (DPMM), identifying two interrelated processes—self-compassion and rumination—through which mindfulness is associated with better mental health. By elucidating these pathways, the study advances theoretical understanding of how mindfulness may simultaneously promote well-being and reduce distress. The model’s validation in a Chinese university sample offers initial cross-cultural support. Future research should adopt longitudinal and cross-cultural designs to explore the stability, directionality, and contextual moderators of these mechanisms, informing both global theory and localized mental health interventions.

## Data Availability

The datasets used and analyzed during the current study are available from the corresponding author on reasonable request.

## Abbreviations

CES-D: Center for Epidemiologic Studies Depression Scale
DPMM: Dual Pathways Mindfulness Model
MAAS: Mindful Attention Awareness Scale
RRS: Ruminative Responses Scale
SCS: Self-Compassion Scale
SEM: Structural Equation Modeling
SWLS: Satisfaction with Life Scale
